# Sarcoidosis-Like Cancer-Associated Granulomatosis: Characteristics and a Case-Control Comparison with Sarcoidosis

**DOI:** 10.3390/jcm10091988

**Published:** 2021-05-05

**Authors:** Jean Pastré, Diane Bouvry, Karine Juvin, Amira Benattia, Isabella Annesi-Maesano, Dominique Valeyre, Hilario Nunes, Dominique Israël-Biet

**Affiliations:** 1 Service de Pneumologie et Soins Intensifs, Assistance Publique-Hôpitaux de Paris Centre, Hôpital Européen Georges Pompidou, 75015 Paris, France; karine.juvin@aphp.fr (K.J.); amira.benattia@aphp.fr (A.B.); dominique.israel-biet@aphp.fr (D.I.-B.); 2 Service de Pneumologie, Assistance Publique-Hôpitaux de Paris, Hôpital Avicenne, 93009 Bobigny, France; diane.bouvry@aphp.fr (D.B.); dominique.valeyre@aphp.fr (D.V.); hilario.nunes@aphp.fr (H.N.); 3 Institut Pierre Louis d’Epidémiologie et de Santé Publique (IPLESP UMRS 1136), Université Pierre et Marie Curie, Paris Sorbonne Cité, INSERM, Epidémiologie des Maladies Allergiques et Respiratoires (EPAR), Hôpital Saint Antoine, 75012 Paris, France; isabella.annesi-maesano@inserm.fr; 4 Université Paris 13, Paris Sorbonne Cité, 93430 Villetaneuse, France; 5 UFR de Médecine, Université de Paris, 75006 Paris, France

**Keywords:** sarcoidosis, cancer, granulomatosis

## Abstract

(1) Background: Systemic granulomatosis developed in a context of malignancy has already been reported. Our objective was to describe the clinical, radiological, functional, biological, and evolutive characteristics of sarcoidosis-like cancer-associated granulomatosis (SLCAG) and to compare them to those of sarcoidosis. (2) Methods: 38 patients with a biopsy-proven SLCAG developed after a diagnostic of malignancy were included. The control group consisted of sarcoidosis patients matched for age, sex, and radiologic stage. Clinical, biological, physiological, radiological, and outcome data were collected. (3) Results: The mean age of SLCAG patients was 51 ± 14 years. They were diagnosed within 15 ± 14 months of the cancer diagnosis (breast cancer most frequently). All SLCAG patients presented a thoracic involvement, extrathoracic locations were observed in 32% of subjects. SLCAG was more often asymptomatic than sarcoidosis (*p* < 0.0001). During follow-up, systemic treatment was less often required in SLCAG than in sarcoidosis (58% vs. 32%, *p* = 0.04 respectively) and SLCAG were characterized by a significantly less severe progression profile according to the Sarcoid Clinical Activity Classification, with a complete recovery more frequent at 5 years (*p* = 0.03). (4) Conclusion: This case-control study shows that SLCAG differs from sarcoidosis with a significantly more benign course. These results might argue for true differences in the physiopathology, which remain to be elucidated.

## 1. Introduction

The association of granulomatous reactions and malignancy has long been known [1,2,3,4,5,6]. That of granulomatosis and lymphoma is even individualized as a sarcoidosis–lymphoma syndrome with distinctive features compared to true sarcoidosis [7,8,9]. In contrast, little is known about the overall baseline characteristics of subjects with a systemic granulomatosis occurring in the context of a solid tumor. In addition, whether these sarcoid-like cancer-associated granulomatosis (SLCAG) behave like sarcoidosis in terms of severity, treatment, and outcome is largely unknown due to the paucity of reports actually comparing these two entities. Based on some differences between their pathophysiologic features [3,7,10,11], we hypothesized that SLCAG and sarcoidosis might have different baseline and outcome characteristics. This point is not trivial since it might have a strong impact on both the prognosis and management of these conditions. Indeed, some studies have underlined the positive impact of sarcoid-like reactions on tumor prognosis [12,13,14,15]. Clinicians should therefore be very cautious in not overtreating these patients, as immunosuppressants used to control the granulomatous inflammation might also result in a decreased tumor control.

In order to actually compare SLCAG and sarcoidosis, we carried out a case-control study of the former, and evaluated its baseline as well as its outcome characteristics and compared them to those of sarcoidosis.

## 2. Patients and Methods

**Figure 1:**
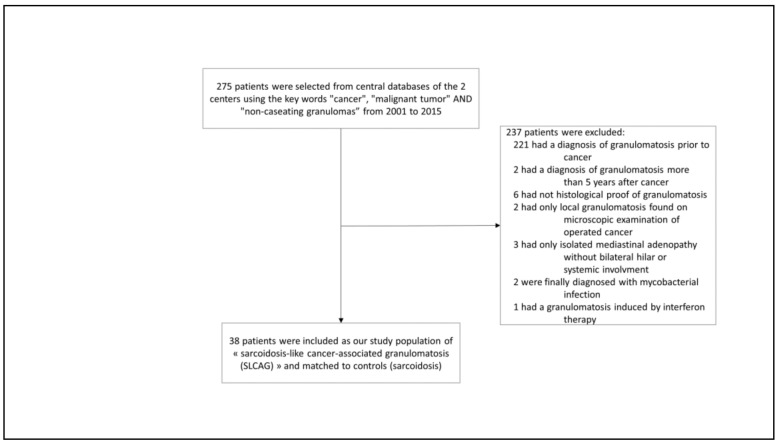
Flowchart of the study.

We performed a retrospective, matched case-control study carried out in two French Competence Centers for Rare Pulmonary Diseases (Hôpital Européen Georges Pompidou, AP-HP, Paris, and Hôpital Avicenne, AP-HP, Bobigny, France), both being tertiary ILD care centers. Institutional Review Board of the French Speaking Society for Chest Medicine. (Société de Pneumologie de Langue Française) approval was obtained for the study n° CEPRO 2019-036. All medical records in these two centers were searched between 2001 and 2015 using the key words “cancer”, “malignant tumor”, and “non-caseating granulomas”. We included in the study all subjects with a biopsy-proven granulomatosis occurring either concurrently or within 5 years after a malignancy. The date of the granulomatous diagnosis defined the baseline. All types of solid tumors or hematological malignancies were included into the study, except lymphomas which have been previously addressed as a specific entity [7,8,9]. Aiming at assessing subjects with a true sarcoid-like disease and not only loco-regional sarcoid-like reactions we included only patients with a bilateral hilar lymphadenopathy and/or with a systemic SLCAG (Figure 1). Non-inclusion criteria were: (1) All other causes of granulomas (particularly infection, drug-induced (especially interferon and immune checkpoints inhibitors), occupational, and environmental). To rule out the former, an extensive infectious work-up had been systematically performed, searching for all bacteria, mycobacteria, parasites and fungi with appropriate stainings and cultures of all the relevant samples (bronchoalveolar lavage and whenever performed, biopsies). (2) Granulomatosis diagnosed before malignancy or more than 5 years after it in order to limit the risk of a fortuitous association between a true sarcoidosis and a cancer. (3) Isolated local granulomatous reactions inside or close to a resected tumor or its draining lymph nodes without any clinical or radiological manifestation. (4) Isolated mediastinal lymphadenopathy.

According to these criteria, we included 38 SLCAG cases (Figure 1) and compared them to a control group of 38 sarcoidosis cases. Within the latter, pulmonary sarcoidosis had been diagnosed according to the American Thoracic Society/European Respiratory Society/World Association of Sarcoidosis and Other Granulomatous Diseases criteria [16,17,18]. Controls were randomly selected according to the time of their first visit, which had to be the closest as possible to that of the SLCAG cases. They were, in addition, matched with SLCAG for age (±5 years) at diagnosis of sarcoidosis, sex, ethnicity, radiological stage, and number of organs involved as a reflection of an overall severity [17].

All subjects had been regularly assessed, at time intervals depending on their clinical status. All medical files of SLCAG and controls were reviewed (JP and DIB) and all clinical, functional, biological (including blood and BAL), radiological, and pathological data at diagnosis, as well as at last follow-up, were collected from the electronical medical records (JP). All chest X-ray radiological staging and thoracic CT scans were reviewed by JP, DIB, and DV. Outcome evaluation focused on clinical status, functional and radiological features if available, as well as on needs for treatment during the follow-up, which had been left at the clinician’s judgement. Disease outcome was also examined using two different tools validated in sarcoidosis: the Sarcoid Clinical Activity Classification (SCAC) [19] with its six progression patterns and, whenever possible, a simplified classification of outcome in four categories derived from the original classification of outcome (COS) [20] and comparable to that used by Pacheco et al. [21]. This simplified COS uses four different outcomes: (1) recovery within 3 years, (2) recovery between 3 and 5 years, (3) persistent granulomatosis activity at 5 years, (4) death. A persistent granulomatosis activity was defined by persistent symptoms and/or need for treatment, radiological progression, or persistent elevated serum angiotensin-converting enzyme.

### Statistical Analysis

The results are expressed as percentage, mean ± standard deviation (SD), or median (range) as appropriate. SLCAG patients and controls were compared using the Fisher’s exact test or non-parametric Wilcoxon paired test when appropriate. *p* values ≤ 0.05 were considered significant.

## 3. Results

### 3.1. Clinical and Laboratory Findings

The SLCAG group consisted of 38 patients aged 51 ± 14 years (mean ± SD), 66% of them being females, 84% Caucasians, and 33% current or ex-smokers. Their malignancy characteristics are shown in Table 1. The most frequent solid tumors were gynecologic. Interestingly, the large majority of these tumors were limited at the time of SLCAG diagnosis, with surgery performed in 76% and with a very high remission rate (76% after a 5-year follow-up) (Table 1). The systemic granulomatosis had been diagnosed within 15 ± 14 months of that of cancer, with a histopathological proof of granulomas in 1, 2, or 3 sites in 35, 2, and 1 patients, respectively. The most common sites of positive samples were thoracic.

Table 2 shows baseline clinical characteristics of patients and controls. Most patients were asymptomatic at the time of diagnosis and the reason for biopsy had been an abnormal imaging, including CT or PET scans. They all presented with bilateral hilar lymphadenopathy (inclusion criteria) whether or not associated with enlarged mediastinal lymph nodes in 36 patients (95%) and/or pulmonary infiltrates in 22 (58%). The median (range) number of organs involved was 1 (1–5) with an extrathoracic involvement in 12 (32%) patients. SLCAG significantly differed from sarcoidosis in several respects. Firstly, they were significantly less often clinically symptomatic (*p* < 0.0001) and when symptomatic they experienced less general (*p* = 0.04), respiratory (*p* = 0.04), and cutaneous (*p* = 0.01) symptoms than controls.

Baseline radiological pulmonary-function tests (PFT) and biological data including bronchoalveolar lavage (BAL) are presented in Table 3. As can be seen, the large majority of patients presented with a chest X-ray in stage 1 or 2. PFT were normal in 25 of 33 (82%) SLCAG cases with only 12% showing a restrictive and 15% an obstructive syndrome. BAL cell differentials showed a lymphocytic (lymphocytes ≥ 20%) alveolitis in 8 (44%) patients with a majority of CD4/CD8 ratios > 3.5. Finally, serum angiotensin-converting enzyme (SACE) levels were higher than the upper limit of normal (ULN) in 16 (50%) patients (mean 1.7 ± 0.6 × ULN, range 1–3.1 × ULN). When compared to control sarcoid patients, none of these baseline features significantly differed between the two groups (Table 3).

### 3.2. Outcome and Treatment

The median (range) follow-up was 32 (4–239) and 73 (0–370) months in SLCAG patients and controls, respectively. Table 4 shows their requirements for treatment of granulomatosis and their outcomes. In terms of PFT evolution (Table 4), 37% of SLCAG patients exhibited a consistent improvement in FVC and 22% a deterioration at the last follow-up. Furthermore, 63% of them also exhibited an improved thoracic imaging during follow-up. Once again, there was no difference between patients and controls in terms of functional and thoracic imaging evolution. In marked contrast, they significantly differed in terms of severity of the disease and of outcome. Indeed, Table 4 shows that while only 32% of SLCAG patients required a treatment during follow-up, 58% of the sarcoid controls did so (*p* = 0.04). In both groups, the treatments used were either steroid alone or associated to hydroxychloroquine or immunosuppressants (methotrexate, azathioprine, TNF inhibitors). Moreover, when evaluated by the Sarcoid Clinical Activity Classification (SCAC), patterns of disease course showed that 53% of SLCAG patients had remained asymptomatic with no need for any treatment vs. only 11% of controls (*p* = 0.0002). Similarly, only 16% of SLCAG patients had been symptomatic with a need for prolonged treatment vs. 53% in controls (*p* = 0.005). Finally, when using the COS assessment, while 41% of SLCAG patients exhibited a complete recovery within 3 years, quite comparably to controls, only 15% presented with a persistent granulomatous activity at 5 years vs. 58% in controls (*p* = 0.03) (Table 4). No death or lung transplantation occurred during follow-up in either group.

## 4. Discussion

Whether granulomatosis developing in a malignant context should be considered as a true sarcoidosis or whether it has some specific features and might represent a different entity is still largely debated. Many case reports or short series in the literature have highlighted the potential development of granulomatous reactions in the context of malignancy [1,2,3,4,5] but very few studies to our knowledge have focused on the characteristics of these patients from the granulomatous disease point of view [6,8,9,22]. This study is the first case-control to actually describe the clinical, radiological, functional, biological, and outcome characteristics of patients with a non-lymphoma sarcoid-like cancer-associated granulomatosis compared to those of sarcoidosis. Our main results show that these two conditions although comparable in terms of histopathology and of systemic distribution markedly differ in terms of outcome. The former is indeed characterized by a more benign course, SLCAG patients being significantly less symptomatic, requiring less treatment and presenting with a more frequently resolved granulomatous activity at 5 years than sarcoid controls.

The frequency of sarcoid-like reactions reported in the literature varies between 2 extremes (0.4% of total mediastinoscopies performed in one center [5] and 26.7% of EBUS-TBNA performed during the follow-up of cancer patients in another center [4]). In most series, however, these frequencies are established around 5.2% and 7.8% [23,24]. In contrast, the prevalence of truly systemic granulomatosis in a malignant context is unknown. Only one series to our knowledge addressed this question, showing that among 227 patients with sarcoidosis, 52 (18.8%) presented with a malignancy [25]. As far as the type of malignancies associated with granulomas is concerned, our own series is in line with the literature showing a large variety of cancer with a predominance of gynecological and particularly breast cancers (17%) [25,26,27,28].

Our aim was to compare SLCAG and sarcoidosis in terms of baseline features and outcome. It is noticeable that both groups exhibited characteristics comparable to what could be expected from the literature regarding sarcoidosis [29,30,31]. For instance, the nature of their main symptoms when present (mostly respiratory and/or general), the sex ratio, the prevalence of pulmonary infiltrates, and of extrathoracic involvement was comparable to literature. However, it is worth underlining that SLCAG patients were slightly older (51 ± 14 yrs, m ± SD) than the classical peak age of sarcoidosis onset [29,30,31], although this remains perfectly in line with most recent epidemiological reports [32]. In addition, and in our own series, these baseline characteristics were similar in the two groups. In contrast, the two conditions markedly differed by several baseline aspects in that SLCAG subjects were more often asymptomatic than sarcoid controls (*p* < 0.0001). Indeed, general, respiratory, and cutaneous conditions were less frequent in SLCAG than in controls, potentially resulting from the fortuitous discovery of granulomas during the systematic follow-up of cancer rather than from clinical signs and/or symptoms. Finally, the most significant differences between SLCAG and sarcoidosis were their outcomes, with SLCAG being significantly more benign than sarcoidosis. Although patients and controls were matched for radiological stages and number of organs involved, SLCAG less frequently required treatment and had better outcomes as defined by SCAC and a modified classification of outcomes (Table 4).

On the other hand, and strikingly, most cancers observed in our series had a very limited baseline extension and a high 5-year remission rate. This is in keeping with previous studies showing a better prognosis of cancer when associated with granulomatous reactions [12,13,14,15] and arguing for a role of these granulomatous reactions in tumor control. Large prospective case control studies are needed to address the very interesting issue of pathogeny and role of granulomas in cancer patients. This was far beyond the scope of our own study.

Altogether, our data might contribute to the concept of SLCAG and sarcoidosis actually being different conditions. SLCAG is most commonly accepted to be due to immune responses towards tumor antigens shed in their close environment or spread around by circulating tumor cells nesting in distant organs rather than to a true multisystemic inflammatory disease [3,7,10,11]. SLCAG and sarcoidosis might also differ in terms of types, production levels, and overall regulation of inflammatory mediators involved. These hypotheses are presently purely speculative, and appropriate studies could help us to get further insights into this complex pathogeny.

Our study has several limitations. Firstly, its retrospective nature made some data unavailable. Secondly, we cannot exclude that some patients included in this study presented with a fortuitous association of a sarcoidosis and a cancer rather than a granulomatosis mediated by the existence of a malignancy, given the prevalence of these two diseases. In addition, some very subtle histopathological differences have been described between sarcoidosis and sarcoid-reactions [33] but they are not assessed in routine diagnostic conditions, and granulomas are most usually described as non-caseating ones in both afflictions. However, to limit this bias, we have excluded from this study all patients with a granulomatosis disease present either before the cancer diagnosis or developing more than 5 years after it (Figure 1). The mean time between cancer and granulomatosis diagnosis being rather short, 15 ± 14 months, this might argue for a non-fortuitous association of both conditions. Another possible limitation is that, due to the systematic pretherapeutic work-up and follow-up of cancer patients, including imaging and prompt biopsy in case of mediastinal lymphadenopathy and/or organ involvement, we cannot exclude a relative overdiagnosis of SLCAG compared to sarcoidosis. However, our study had focused neither on SLCAG prevalence nor on its underlying tumor characteristics, but on SLCAG clinical characteristics and outcome in comparison with true sarcoidosis. In this respect, we think that the case-control design of this study with matching for age, sex, ethnicity, radiological stage, and number of organs involved for all patients might have limited overinterpretations of true differences between SLCAG and sarcoidosis. Finally, although some chemotherapeutic agents are known to induce hypersensitivity and/or granulomatous reactions [1,34], we have tried our best to rule out iatrogenic reactions by a careful examination of all medical files based upon clinical history, follow-up, and literature-based data (https://pneumotox.com, accessed on 6 April 2021) in addition to having excluded all patients treated with the most frequent granuloma inducers in this context (i.e., interferon and immune check point inhibitors).

## 5. Conclusions

This study of SLCAG vs. sarcoidosis highlights differences between these two entities with a significantly more benign course of the former. Clinicians in charge of such patients should be aware of this more benign prognosis and more frequent spontaneous resolution as well as of the generally accepted positive effects of granulomatous reactions on the tumor control when designing therapeutic strategies.

## Figures and Tables

**Table 1:** Demographics and malignancy characteristics of SLCAG patients.

		SLCAG Patients
*N*		38
Age, years (mean ± SD)		51 ± 14
Females, *n* (%)		25 (66%)
Caucasian/black ethnicity, *n* (%)		32 (84%)/4 (11%)
Smokers, *n* (%, mean pack-years)		12 (33%, 30)
Site of malignancy *n* (%)	Breast	7 (17%)
	Gynecologic (endometrial or ovarian)	5 (13%)
	Lung	4 (11%)
	Colorectal	4 (11%)
	Head and neck	4 (11%)
	Renal	3 (8%)
	Prostatic	2 (5%)
	Skin (melanoma)	2 (5%)
	Others	7 (19%)
Cancer stage at diagnosis *n* (%)	Limited	21 (55%)
	Regional lymph nodes extension	5 (13%)
	Metastatic extension	0
	Not available or not applicable	12 (32%)
5 years remission rate, *n* (%)		29 (76%)

SLCAG: sarcoidosis-like cancer-associated granulomatosis.

**Table 2:** Baseline clinical characteristics of SLCAG and sarcoid patients.

		SLCAG	Sarcoidosis Controls	*p* Value
Symptomatic, *N* (%)		13 (34%)	32 (86%)	<0.0001
Symptoms *n* (%)	General	4 (11%)	12 (33%)	0.04
	Respiratory	11 (29%)	21 (58%)	0.04
	Cutaneous	1 (3%)	9 (25%)	0.01
	Ophthalmic	0	4 (11%)	0.11
	Löfgren syndrome	0	3 (8%)	0.24
Organs involved *n* (%)	Thorax	38 (100%)	38 (100%)	
	Bilateral lymph nodes with or without mediastinal ones	36 (95%)	38 (100%)	0.49
	Thorax alone	26 (68%)	27 (71%)	1.0
	Pulmonary infiltrates	22 (58%)	23 (60%)	1.0
	Extrathoracic	12 (32%)	11 (29%)	1.0
	Extrathoracic lymphadenopathy	9 (24%)	3 (8%)	0.11
	Liver	4 (10%)	2 (5%)	0.67
	Heart	3 (8%)	2 (5%)	1.0

SLCAG: sarcoidosis-like cancer-associated granulomatosis.

**Table 3:** Radiological, functional, and biological characteristics of SLCAG and sarcoidosis patients.

		SLCAG	Sarcoidosis Controls	*p* Value
X-ray staging *n* (%)	1	18 (48%)	17 (45%)	
	2	14 (37%)	15 (39%)	
	3	4 (10%)	2 (5%)	
	4	2 (5%)	3 (8%)	
PFT *n* (%)	*n*	33	38	
	TLC < 80% pred	4 (12%)	9 (24%)	0.24
	FVC < 80% pred	6 (18%)	9 (24%)	0.77
	FEV_1_/FVC < 70%	5 (15%)	5 (13%)	1.0
	TLCO < 80% pred	22 (66%)	31 (82%)	0.18
BAL	*n*	18	30	
	Lymphocyte count, mean ± SD, %	21 ± 17	27 ± 20	0.08
	Lymphocyte count ≥20%, *n* (%)	8 (44%)	17 (57%)	0.55
	CD4/CD8 T-cell ratio >3.5, *n* (%)	6/10 (60%)	10/16 (62%)	1.0
Increased SACE, *n* (%)		16/32 (50%)	17/37 (46%)	0.81

SLCAG: sarcoidosis-like cancer-associated granulomatosis; PFT: pulmonary-function tests; TLC: total lung capacity; FVC: forced vital capacity; FEV1: forced expiratory volume in 1s; TLCO: transfer factor of the lung for carbon monoxide; SACE: serum angiotensin-converting enzyme; BAL: bronchoalveolar lavage.

**Table 4:** Treatment and outcome of SLCAG patients and sarcoidosis controls.

		SLCAG	Sarcoidosis Controls	*p* Value
Follow-up, Median (Range), Months		32 (4–239)	73 (0–370)	0.12
PFT change from baseline at last FU, n/available (%)	FVC improvement *	10/27 (37%)	8/28 (29%)	0.57
	FVC deterioration ^†^	6/27 (22%)	5/28 (18%)	0.75
	TLCO improvement *	8/27 (30%)	11/33 (33%)	0.79
	TLCO deterioration ^†^	4/27 (15%)	5/33 (15%)	1.0
Treatment, *n* (%)		12 (32%)	22 (58%)	0.04
Corticosteroids, *n* (%)		9 (24%)	19 (50%)	0.03
Others, *n* (%)		9 (24%)	13 (34%)	0.45
SCAC *n* (%)	*n*	38	36	
	1—asymptomatic—no treatment	20 (53%)	4 (11%)	0.0002
	2—asymptomatic—treatment ≤12 months	0	1 (3%)	0.49
	3—asymptomatic—treatment >12months	4 (10%)	0	0.11
	4—symptomatic—no treatment	6 (16%)	13 (36%)	0.06
	5—symptomatic—treatment ≤12 months	2 (5%)	1 (3%)	1.0
	6—symptomatic—treatment >12 months	6 (16%)	17 (53%)	0.005
Classification of outcome *n*/available (%)	(1) Recovery within 3 years	12/29 (41%)	13/33 (39%)	1.0
	(2) Recovery between 3 and 5 years	0	0	
	(3) Persistent GD activity signs at 5 years	2/13 (15%)	11/19 (58%)	0.03
	(4) Death	0	0	

SLCAG: sarcoidosis-like cancer-associated granulomatosis; PFT: Pulmonary function tests; FVC: forced vital capacity; TLCO: transfer factor of the lung for carbon monoxide; * Improvement is defined as an increase >5% of FVC and >15% of TLCO compared to baseline; ^†^ Deterioration is defined as a decrease >5% of FVC and >15% of TLCO compared to baseline; SCAC: Sarcoid Clinical Activity Classification; GD: granulomatous disease.

## Data Availability

Data are available on reasonable request.
